# Regulation and financing of prenatal screening and diagnostic tests for fetal anomalies in Europe

**DOI:** 10.1093/eurpub/ckac130.046

**Published:** 2022-10-25

**Authors:** I Reinsperger, I Zechmeister-Koss

**Affiliations:** Austrian Institute for HTA, Vienna, Austria

## Abstract

**Background:**

Pregnant women frequently use prenatal screening and diagnostic tests to detect fetal structural and chromosomal anomalies; however, the regulation and financing of these examinations differ substantially across countries. In this paper we focus on the provision, financing and setting of the following tests in selected European countries: First Trimester Screening (FTS)/Combined Test (CT), Non-invasive Prenatal Test (NIPT), second-trimester ultrasound screening for fetal anomalies, amniocentesis/chorionic villus sampling.

**Methods:**

We chose 6 European countries that differ in various criteria (e.g., health/insurance system, geographical location) to illustrate the range of possible regulations and forms of funding: Germany, Switzerland, Netherlands, United Kingdom, Norway, Italy. We conducted a comprehensive hand search and consulted experts from the 6 countries using a questionnaire.

**Results:**

The results are based on 11 completed expert questionnaires and 22 published sources. The heterogeneity of the provision, regulation and financing of the tests concerns in particular the choice of the first-line screening test for fetal trisomies (FTS/CT, NIPT or the identification of risk factors), the implementation of the NIPT, the reimbursement of the tests, the uptake of the examinations, but also the professional groups responsible for antenatal care (midwives, gynaecologists). There are some similarities between countries, e.g., concerning the provision and financing of invasive tests and of the ultrasound screening for fetal anomalies in the second trimester.

**Conclusions:**

The results highlight the significant heterogeneity between European countries regarding prenatal screening and diagnostic testing for fetal anomalies. Due to the many ethical aspects of the topic, a broad societal discourse with the relevant interest groups and stakeholders seems to be necessary. Decision-makers should pay particular attention to high-quality and non-directive counselling.

**Key messages:**

- European countries take different approaches to offer and fund prenatal screening and diagnostic tests for fetal anomalies.
- The implementation of screening strategies should focus in particular on the importance of high-quality counselling to enable informed decision-making.

